# The Mycorrizal Status in Vineyards Affected by Esca

**DOI:** 10.3390/jof7100869

**Published:** 2021-10-16

**Authors:** Lucia Landi, Renzo Foglia, Sergio Murolo, Gianfranco Romanazzi

**Affiliations:** Department of Agricultural, Food and Environmental Sciences, Marche Polytechnic University, Via Brecce Bianche, I-60131 Ancona, Italy; l.landi@univpm.it (L.L.); r.foglia@univpm.it (R.F.); s.murolo@univpm.it (S.M.)

**Keywords:** droplet digital PCR, esca, grapevine roots, mycorrhizal non-vital staining, native AMF, operational taxonomic units (OTUs)

## Abstract

In this work we analyzed the relationship among native arbuscular mycorrhizal fungi (AMF) and vine roots affected by esca, a serious grapevine trunk disease. The AMF symbiosis was analyzed on the roots of neighboring plants (symptomatic and asymptomatic to esca) in 14 sites of three vineyards in Marche region (central–eastern Italy). The AMF colonization intensity, identified by non-vital staining, showed higher value in all esca symptomatic plants (ranging from 24.6% to 61.3%) than neighboring asymptomatic plants (from 17.4% to 57.6%). The same trend of Glomeromycota phylum abundance was detected by analyzing fungal operational taxonomic units (OTUs) linked to the AMF community, obtained by amplicon high throughput analysis of ITS 1 region. Overall, the highest amount of OTUs was detected on roots from symptomatic plants (0.42%), compared to asymptomatic roots (0.29%). Specific primer pairs for native *Rhizophagus irregularis* and *Funneliformis mosseae* AMF species, were designed in 28S rRNA and large subunit (LSU) ribosomal RNA, respectively, and droplet digital PCR protocol for absolute quantification was set up. A higher number of DNA copies of both fungal species were detected more frequently in symptomatic than asymptomatic vines. Our study suggests a relationship between esca and native AMF in grapevine. These results underline the importance of native rhizosphere microbial communities for a better knowledge of grapevine esca disease.

## 1. Introduction

Esca, together with Eutypa dieback and Botryosphaeria dieback, are considered the most destructive trunk diseases of grapevine, and are a rapidly growing concern in all wine producing countries [1]. This disease involves several xylem-inhabiting fungi [2,3,4,5,6,7]. The general symptoms are expressed on the one hand at the foliar level by discoloration and drying and on the other hand at wood level through sectorial necrosis with the presence of brown streaking or cankers [8]. Characteristic symptoms of grapevines affected by these diseases are sunken necrotic root lesions with a reduction in root biomass and root hairs [9]. As a result, the symptoms produced by esca are detrimental to the resilience of the wine-growing heritage [1]. Since the trunk disease’s pathogens are mainly soilborne [1,10], a helpful role in plant health management may be played by rhizosphere microorganisms, such as mycorrhizal fungi. Arbuscular mycorrhizal fungi (AMF, phylum Glomeromycota, class Glomeromycetes) [11], are the most common obligate biotrophic symbiotic fungi associated with plant species. More than 80% of plant species in natural conditions are mycorrhized. This exceptionally ancient (>450 million years) and coevolutionary relationship is considered the key factor in early plant colonization of land and has also been verified to be generally beneficial to both partners [11,12]. The AMF are completely dependent on their host’s carbon source, then, as mutual exchange, the plant receives additional nutrients and improved water relations [12,13,14]. Furthermore, in addition to this nutritional function, AMF field and pot inoculation can enhance plant tolerance to both biotic and abiotic stresses including drought, salinity, herbivory, temperature, metals, and diseases, relative to their non-mycorrhizal counterparts in experimental studies [15]. Several mechanisms whereby AMF could cause pathogen protection are known, including changes in root architecture, improvement of nutrient status, competition for infection sites or activation of plant defence mechanisms, including the upregulation of the antioxidant system, modifications in the phytohormone profile and secondary metabolites [16,17,18]. This multifunctional ability of the partner fungi has led to the development and applications of mycorrhizal inoculants as biofertilizers in agriculture. Mycorrhizal inoculation has been applied for decades to promote better plant growth for various crop plants [19]. Several studies point out the higher efficiency of native AMF inoculum, starting from native soils as an alternative to commercial formulates [20,21,22,23]. A different role could be played by the nontarget native mycorrhiza, naturally present in the soil. The native AMF which forms multispecies symbiont communities in the same host root, are well adapted to the local environment [24]. Various agricultural management practices modify the native AM fungal community qualitatively and quantitatively [25]; however, there is not enough information about the relationship of mycorrhizal native species, already established in the plant, and plant diseases. Concerning the grapevine plants, they are remarkably dependent on mycorrhizae, since this plant has low-density roots and few root hairs. In vineyards, the AMF communities mainly belong to the Glomerales order, phylum Glomeromycota [26,27]. They are highly influenced by the soil characteristics but also, to a smaller extent, by the host plant development stage and cultivars [28,29]. Concerning the AMF external inoculum effect on trunk disease, different results were observed, as there was an increase in tolerance to grapevine disease [30], but an enhancement of in the number of pathogens present was also observed [31]. Additionally, although the key role of AMF in plant productivity and ecosystem is undisputed [27,32], different relationships among mycorrhizal and pathogens detected on plants, underline the complexity of plant–AMF–pathogen interactions relationship [33,34]. Until now, the relationship between native AMF community and esca diseases was unexplored. Therefore, to establish the impacts that esca has on the whole beneficial AMF—plant symbiosis, one of the primary approaches is to quantify the total AMF colonization on roots from asymptomatic or symptomatic grapevines. Usually, non-vital staining of mycorrhizal colonization [35], followed by microscopic investigation, has provided reliable data on the degree of root colonization. This is an easy and fast method for analyzing the whole root’s mycorrhizal status, including the presence and distribution of key features such as arbuscules, processing a large number of samples but without discriminating the AMF species [33,36]. Recently, the omics approach has allowed researchers to quantify the abundance of mycorrhiza in both plant roots and soil [17,37,38]. For high throughput sequencing, the operational taxonomic units (OTUs) technique was used in several works according to rRNA (SSU rRNA) [39], or internal transcribed spacer (ITS) region, this last as universal barcode for fungi marker genes clustered into OTUs according to nucleotides’ similarity [40]. For species-specific detection and absolute quantification of single sequence, the droplet digital PCR (ddPCR) is widely used. This method is now often used as alternative to quantitative real-time PCR (qPCR) [41]. Recently, it was also applied for *Rhizophagus irregularis* copy number quantification [32]. ddPCR s based on the amplification of single target DNA molecules in many separate droplets (water-in-oil emulsion) resulting in a positive–negative call for every droplet and greater amenability to multiplexed detection of target molecules [42]. With respect to qPCR, ddPCR offers the advantage of direct and independent quantification of DNA without standard curves and reduction of numbers of technical replicates [43].

The goal of this work was to investigate the relationships among of AMF native root symbionts and esca disease. To achieve this aim, several approaches were used: (i) evaluating total AMF colonization using non-vital Trypan blue (TB) staining; (ii) analyzing the global abundance of Glomeromycota phylum according the OTUs with ITS primers; (iii) developing and validating the ddPCR identification and quantification of *R. irregularis* and *Funneliformis mosseae* selected as marker species in the vineyard soils among Glomerales order.

## 2. Materials and Methods

### 2.1. Experimental Trials

The experiment was performed from January to July 2017 in the three commercial vineyards, located in Ancona district, Marche region, in central-eastern Italy. In detail:

Vyn_1—The vineyard, located in Castelplanio (AN), 43°50′30″ N–13°09′50.3″ E, was planted in 2004. The grape cultivar was ‘Verdicchio’ grafted onto Kober 5BB (*Vitis berlandieri* × *V. riparia*) rootstock. The soil has a medium texture tending to be melted, with a scarce amount of organic matter. The morphology of the land is hilly, and the exposure of the vineyard is south/west. The roots of the selected vine plants were collected in January. 

Vyn_2—The vineyard, located in San Biagio of Osimo (AN), site at 43°31′09″ N–13°28′47″ E at 463 meters above sea level (m a.s.l.), was planted in 2002. The grape cultivar was ‘Verdicchio’ grafted onto Kober 5BB rootstock. The soil has a medium texture tending to clay, fresh, fertile, deep and mainly grassy. The roots from the selected grapevine plants were collected in May. 

Vyn_3—The vineyard, located in Osimo (AN), site at 43°29′81″ N–13°26′12.83″ E, at (m a.s.l.) was planted in 2002. The grape cultivar was ‘Chardonnay’, grafted onto Kober 5BB rootstock. The vineyard is exposed to the south/east. The soil has a medium texture, with a high organic substance. Usually, the soil is ploughed both in the row and along the rows, with elimination of any infesting flora. The roots from the selected grapevine plants were taken in July.

The investigated vineyards were not irrigated, the fertilizers were distributed in winter, and an additional green pruning was applied in spring and summer, as are the normal practices for the area. In all vineyards, an integrated pest management program was applied to control the main fungal diseases (downy mildew, powdery mildew, gray mold) and pests (moths).

### 2.2. Collected Samples 

In each vineyard, fourteen sites were randomly selected. From each site, one symptomatic plant (I) and the neighboring asymptomatic plant (A) were selected for the study (Supplementary Figure S1). In detail, four plants for the vineyards Vyn_1 (plants A1, I1; A2, I2; A3, I3; A4, I4 from sites 1, 2, 3 and 4, respectively) and Vyn_2, (plants A5, I5; A6, I6; A7, I7 and A8, I8 from sites 5, 6, 7 and 8, respectively) and six for vineyard Vyn_3 (plants A9, I9; A10, I10; A11, I11; A12, I12; A13, I13 and A14, I14; from sites 9, 10, 11, 12, 13 and 14). A total of 28 plants were analyzed: 14 plants showing “leaf tiger-stripes” (symptomatic vines) and 14 asymptomatic plants. From each plant, the roots were collected at a depth of about 20 cm. A portion of the terminal root system from each plant was randomly selected and washed with tap water. A total of 8–10 g from each plant were collected, half of which were stored in acetic acid:ethanol (50:50) solution for total non-vital staining. Half of the roots were stored at −20 °C for the following DNA extraction. 

### 2.3. Total Native AMF Assessment Using Non-Vital Staining and Light Microscopy 

For assessing the total AMF symbiosis, from each plant, secondary roots were collected from different points of plant’s roots system. Then, 50 sections of 1 cm-long pieces were randomly chosen and analyzed by non-vital staining with Trypan Blue (TB) [44]. After clearing the root samples in 10% KOH solution for 20 min at 80 °C, the roots were stained with 0.05% (*w/v*) TB in lactoglycerol (lactic acid:glycerol:H_2_O, 1:1:1, *v/v/v*) for 20 min at 80 °C. Stained roots were observed with a light microscope, Nikon Eclipse E600 microscope (Nikon, Tokyo, Japan). The degree of mycorrhizal colonization of each root segment and the abundance of arbuscules were estimated as described by Trouvelot et al. [45]. In detail, 5 classes described the percentage of AMF colonization intensity 0 = 0%; 1 ≤ 1%; 2 ≤ 10%; 3 ≥ 11% < 50%; 4 ≥ 51% < 90%; 5 ≥ 91%. To estimate the abundance of arbuscules, 4 different classes were used: A0 = 0; A1 = few arbuscules, (from N° 0 to N° 5 for root segments); A2 = frequent (from N° 6 to N° 20 for root segments); A3 = abundant (>of N° 20). The MYCOCALC software was used to calculate the frequency of mycorrhiza in the root system (F%), colonization intensity (M%) and abundance of arbuscules (A%) parameters using the following equations: F% = (l_m_/l_t_) * 100; M% = (95n_5_ + 70n_4_ + 30n_3_ + 5n_2_ + n_1_)/l_t;_ A% = a% * 0.01 M%. Where: l_m_ is the total number of root segments in which mycelium were found; l_t_ is the total number of the examined segments; n_5_ –n_1_ is the total number of root segments in which the degree of colonization intensity by mycorrhizal structures was 5-1, (n5 = number of fragments rated 5; n4 = number of fragments 4, etc.); a% is related to absolute abundance of arbuscules for the segments in which arbuscules were present (for more details: https://www2.dijon.inrae.fr/mychintec/Mycocalc-prg/download.html, accessed 15 September 2021).

### 2.4. Molecular AMF Native Detection and Quantification 

#### 2.4.1. DNA Extraction

For each sample, two subsamples were obtained. From each subsample, 2 g of pooled roots ground in liquid nitrogen, then 200 mg of pulverized tissues were collected and put in 2 mL microcentrifuge tube for total DNA extraction. The CTAB (cetyl trimethyl ammonium bromide) procedure [46] modified by Landi et al. [47] was used. DNA purity and quantity were estimated by BioPhotometer plus (Eppendorf Inc., Westbury, NY, USA). Preliminary test to detect two of the main pathogens linked to esca as *Phaeomoniella chlamydospora* and *Phaeoacremonium ultimum*, performed by PCR [48], was confirmed only on symptomatic plants (data not shown).

#### 2.4.2. Overall Quantification of Glomeromycota with High-Coverage ITS Primers 

Total DNA extracted from all asymptomatic and symptomatic root samples, was amplified in total volume 25 μL using the primer pair ITS1F_KYO2 (5′TAGAGGAAGTAAAAGTCGTAA 3′) and ITS2_KYO2 (5′TTYRCTRCGTTCTTCATC 3′) [49], able to amplify the ITS 1 region. PCR was conducted using under a temperature profile of 94 °C for 4 min, followed by 35 cycles at 94 °C for 30 s, 56 °C for 30 s, and 72 °C for 20 s, followed by 72 °C for 7 min. The concentration of MgCl_2_, dNTPs, PCR primers and the template DNA in the reaction buffer were 1.5 mM, 200 mM, 0.5 mM and 1 ng/μL, respectively. The amplified samples were checked on agarose gel, then the amplicons of asymptomatic and symptomatic samples were, respectively (or separately) pooled, obtaining two different samples. The two samples were purified using Wizard^®^ SV Gel and the PCR Clean-Up System (Promega Madison, WI, USA), then quantified with the Biophotometer (Eppendorf), in order to estimate the threshold of quantity (60 ng/μL; at least 500 ng) and quality (260/280 > 1.8; and at 260/230 in range of 1.7). 

#### 2.4.3. DNA Sequencing and Bioinformatics Analysis

Fungal ITS1 amplicon pools related to symptomatic and asymptomatic plants were sent to GENEWIZ, Inc. (South Plainfield, NJ, USA), which provided library preparations, Illumina MiSeq sequencing and data analysis according to the manufacturer’s instructions of GENEWIZ Amplicon-EZ technology. Sequences were grouped into operational taxonomic units (OTUs) using the clustering program VSEARCH (1.9.6) against the UNITE ITS database (https://unite.ut.ee/; accessed 15 September 2021) pre-clustered at 97% sequence identity. The Ribosomal Database Program (RDP) classifier was used to assign taxonomic category to all OTUs at confidence threshold of 0.8. The RDP classifier uses the UNITE ITS database, which has taxonomic categories predicted to the species level.

### 2.5. Rhizophagus Irregularis and Funneliformis Mosseae Absolute Quantification 

#### 2.5.1. Primer Selections and Validation

Specific primers were selected according to 28S rRNA and large subunit (LSU) ribosomal RNA genes for *R. irregularis* (National Center for Biotechnology Information, NCBI cod. HF968988.1) and *F. mosseae* (NCBI cod. FN377865.1), respectively, using Primer3web version 4.1.0. (http://bioinfo.ut.ee/primer3-0.4.0/, accessed 15 September 2021). The primers named RI/f (5′-GGCGTTATTGTCGCACCTAT3′), and RI/r (5′-CCTTGGTTTTTCAAGGGTCA-3′), that amplify a 248 bp PCR fragment, were developed for *R. irregularis* species and primers named FM/f (5′-CCTATGGATCCCCCTTTTGT3′) and FM/r (5′-AGATGCTGCAGAAGGCAAAT3′), that amplify a 190 bp PCR fragment, were developed for *F. mosseae* species.

#### 2.5.2. qPCR Assay

Before using the primers in ddPCR technology, for absolute quantification of *R. irregularis* and *F. mosseae* in the root samples, their ability and specificity to detect the species present in the root samples was validated according to qPCR protocol followed by amplicon sequencing analysis. The qPCR analysis was performed according the Landi et al. [47] protocols using the primers pair RI/f- RI/r and FM/f-FM/r. 

To validate the homology with the AMF species, the sequence analysis of qPCR amplicons obtained from RI/f-r, primers by I1 and A5 plant roots (named S1_V1 and S5_V2 samples) and FM V/f-r primers by I4 and A10 plant roots (named S4_V1 and S10_V3 samples) were performed. The fragments were sequenced by Genewiz (Hope End, Takeley, UK) and subjected to bioinformatic analysis. Sequence similarity searches were performed using Blast analysis in NCBI. 

After sequencing and qPCR validation, a serial dilution of qPCR fragments amplified from root samples generating a qPCR standard curve was performed. Both the limits of detection (LOD) estimated from analysis of number of positive replicates, [50], and possible inhibitors of the root’s matrix, were estimated. In detail, four-point ten-fold serial dilution of *R. irregularis* qPCR fragment S1_V1 (from 3.5 × 10^−8^ to 3.5 × 10^−11^ ng/reaction) and *F. mosseae,* qPCR fragment S10_V3 (from 2.8 × 10^−8^ to 2.8 × 10^−11^ ng/reaction) were amplified alone or spiked with DNA from *Quercus ilex*, holm oak, AMF nonhost roots (50 ng, and 5 ng/reaction). In addition, several dilutions (100, 50, 5, 0.5 ng) of DNA from roots of esca symptomatic plant I10 and asymptomatic plant A7 were also analyzed (the analysis of these samples was carried out after mixing the DNA of the two respective subsamples). DNA from grapevine leaf tissue was included as negative control. The same sample dilutions were tested in the ddPCR setup, with the exception of the spiked with 5 ng/reaction of DNA from holm oak. 

#### 2.5.3. ddPCR Assay

The ddPCR assay was performed using QX200 Droplet Digital PCR system (Bio-Rad Laboratories, Hercules, CA, USA). With the aim to setup and validate the ddPCR assay, serial dilution of both, *R. irregularis* and *F. mosseae* 28SRNA PCR fragment alone or combined with DNA from AMF holm oak nonhost roots, and of the I10 and A7 grapevine roots samples, previously described and analyzed on qPCR assay, were tested. The analysis was carried out in 20 μL of reaction mixture including 1× QX200 ddPCR EvaGreen supermix (Bio-Rad), 0.3 or 0.5 μM each primer. Each 20 μL reaction mixture was transferred into the DG8 cartridge. Next, Droplet Generation Oil (Bio-Rad) was added to the cartridge which was placed into the QX200 Droplet Generator™ (Bio-Rad). After droplet generation, the mixtures were carefully transferred to a ddPCR™ 96-well PCR plate (Bio-Rad) after which the plate was sealed at 180 °C using PX1™ PCR plate sealer (Bio-Rad). The amplification was performed in the thermal cycler, ICycler (Bio-Rad), with a ramp rate of 2 °C/s with the following protocol: 95 °C for 5 min followed by 40 cycles of denaturation at 95 °C for 30 s and 58 °C for 1 min. The enzyme was deactivated at 4 °C for 5 min followed by 90 °C for 5 min. Droplets were read in a QX200 Droplet reader (Bio-Rad) after which the ddPCR data were analyzed using QuantaSoft Version 1.6.6. The script analyzed the data of the signals exported from the QuantaSoft software, with its automatic threshold defined or with a selected, manually defined, threshold applied. This incorporates the calculation of the basic parameters of the ddPCR (i.e., concentration, mean amplitudes of positive and negative droplets); the mean copies per partition and the total volume of the partitions measured, as defined by the digital MIQE guidelines [51]. Two positive droplets were enough to determine a sample as positive, and only the reactions with more than 10,000 accepted droplets were used for analysis. After protocols setup, 50 ng of DNA from symptomatic and asymptomatic root samples were analyzed using 0.3 μM each primer.

### 2.6. Statistical Analysis

Data sets according to AMF colonization of symptomatic and asymptomatic plants, detected by non-vital staining, were analyzed according to Student’s paired *t*-test. Differences were considered significant when *p* < 0.01. The same data sets were tested for correlations using the Pearson’s coefficient (r) *p* < 0.05. The difference among AMF symbiosis in the esca symptomatic and asymptomatic plants according to the vineyards were statistically analyzed (Vyn_1, n = 4; Vyn_2, n = 4; Vyn_3 n = 6). The arcsin square root transformation was used to normalize the percentage ratios data prior to statistical analysis. The data set of AMF colonization was subjected to one-way ANOVA for mean comparisons, standard deviation (SD) and significant differences calculated according to Duncan’s Multiple Range Test, *p* < 0.05. 

For qPCR set-up using I10 and A7 root samples and spiked samples (see Table 1 and Supplementary Tables S1–S3) (three replicates in three independent experiments, n = 9); and ddPCR set-up (mixed subsample in two independent experiments, n = 2) the SD was performed. Linear regression analysis in ddPCR investigation between copies of target genes/ng of root DNA, or ng of qPCR amplicon were analyzed using Excel 2010. Data sets according to ddPCR absolute quantification of target genes by *R. irregularis* and F. mosseae for each plant were statistically analyzed (two independent experiments for each subsample, n = 4). Data set of ddPCR absolute quantification was subjected to one-way ANOVA for mean comparisons, standard deviation (SD) and significant differences calculated according to Duncan’s Multiple Range Test, *p* < 0.05.

## 3. Results

### 3.1. Total Native AMF Assessment Using Non-Vital Staining and Light Microscopy

**Figure 1:**
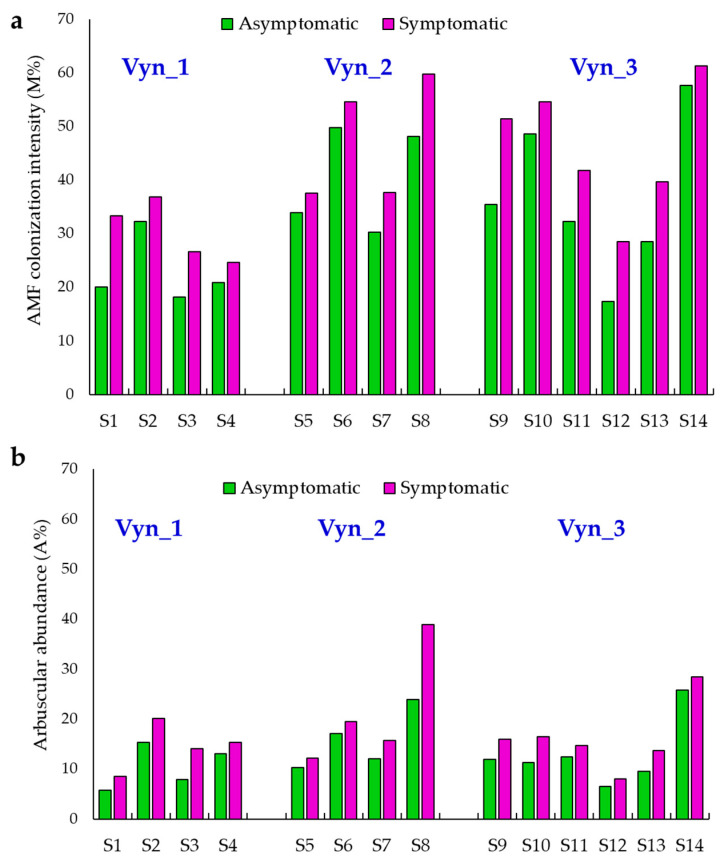
AMF mycelium colonization (M%), (**a**), and arbuscular abundance (A%) (**b**), detected by non-vital staining from each asymptomatic and contiguous esca symptomatic plants from each site (S) individuate in the vineyards (Vyn_1, Vyn_2 and Vyn_3).

Related to non-vital staining, native AMF colonization was detected in all analyzed root samples of both esca symptomatic and asymptomatic plants with a frequency (F%) of around of 100%. Relating to each of the 14 sites identified in the vineyards, all the esca symptomatic plants showed higher significative native mycorrhizal colonization related to both mycelium colonization (M%) (*p* < 0.0001, Student’s paired *t*-test) and arbuscular abundance (A%), (*p* = 0.0005, Student’s paired *t*-test) than the neighboring asymptomatic plants (Figure 1a,b). In the symptomatic, plants the M% ranged from 24.6%, detected in the symptomatic plant I4, to 61.3%, in I14; while in the asymptomatic plants the M% ranged from 17.4%, detected in A12, to 57.6%, detected in A14 (Figure 1a). The A% abundance in symptomatic plants ranged from 8.1%, related to plant I1, to 38.9%, detected in plant I8, while in asymptomatic plants the A% ranged from 5.7%, in A1, to 25.8% in plant A14 (Figure 1b).

**Figure 2:**
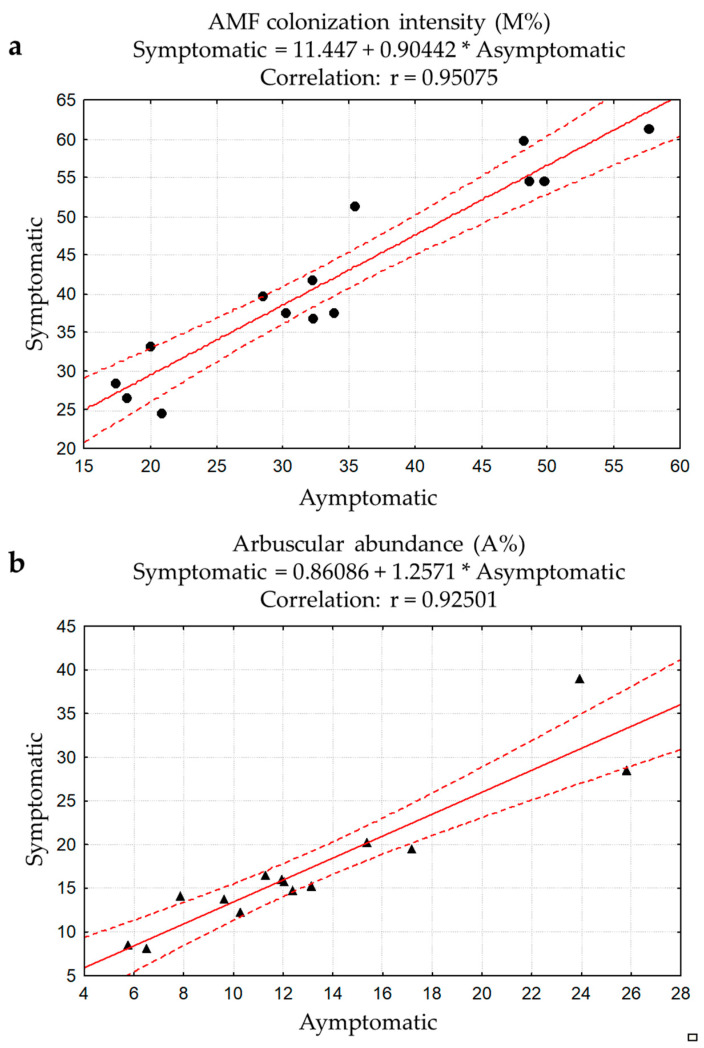
Correlation related to AMF mycelium colonization (**a**) and arbuscular abundance (**b**) detected by non-vital staining in asymptomatic and nearby esca symptomatic plants. Pearson’s coefficient (r) *p* < 0.05.

Positive correlation among esca symptomatic and asymptomatic plants of both AMF mycelium colonization (Figure 2a) and arbuscular abundance was detected (Figure 2b).

**Figure 3:**
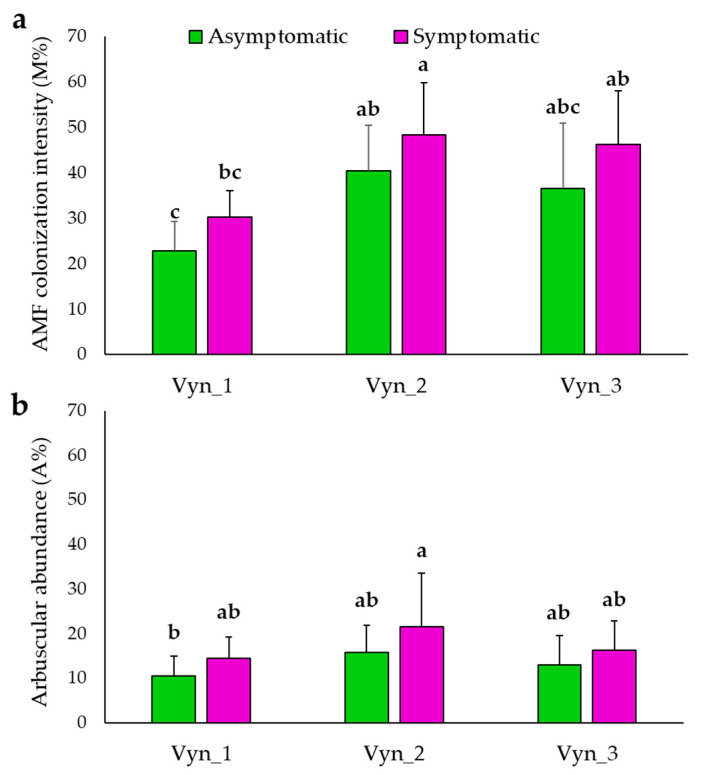
AMF mycelium colonization (% M) (**a**) and arbuscular abundance (% A) (**b**) detected by non-vital staining of roots of esca symptomatic and asymptomatic grapevine plants collected in three different vineyards (Vyn_1, Vyn_2 and Vyn_3). For each trait, means followed by at least one common letter are not significantly different, according to Duncan’s Multiple Range Test (*p* < 0.05). The data were normalized according to the arcsine square root.

The average mycorrhization value of the plants analyzed for each vineyard was also analyzed. For each vineyard, the average M% ranged from 30.3% (Vyn_1) to 48.4% (Vyn_2) (Figure 3a), while the A% ranged from 14.5% (Vyn_1) to 21.6% (Vyn_2) (Figure 3b). In the asymptomatic plants, the M% value ranged from 22.8% (Vyn_1) to 40.5% (Vyn_2) (Figure 3a), while the A% value ranged from 10.5% (Vyn_1) to 15.8% (Vyn_2) (Figure 3b). The Vyn_3 showed intermediate values. Not significant differences in native mycorrhizal infection were recorded between asymptomatic and symptomatic plants in each vineyard. 

### 3.2. Overall Quantification of Glomeromycota with High-Coverage ITS Primers

**Figure 4:**
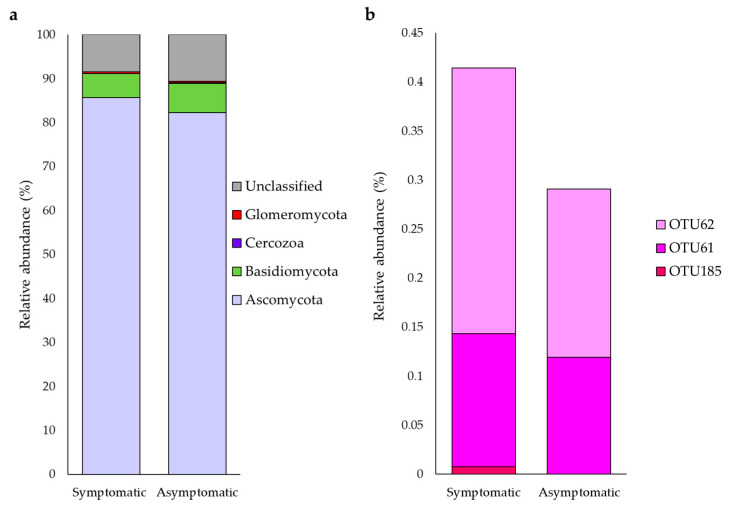
Phylum composition of the fungal community (**a**) and change in abundance of OTU assigned to Glomeromycota phylum (**b**) related to AMF, associated with pool of DNA from asymptomatic and symptomatic grapevine to esca.

A total of 66,352 quality reads were obtained from asymptomatic and symptomatic pool samples, grouped into 241 OTUs for taxonomic affiliation with Ascomycota, Basidiomycota, Cercozoa, and Glomeromycota, at phylum level. Other different OTUs sequences generically assigned to fungi kingdom were also detected (Figure 4a). From the bioinformatic analysis, 275 sequences detected in roots from symptomatic plants, and 193 sequences, detected in roots from asymptomatic plants, have been assigned to three OTUs defined as phylum Glomeromycota (Figure 4b). 

### 3.3. Rhizophagus Irregularis and Funneliformis Mosseae Absolute Quantification 

#### 3.3.1. Primer Selection and Validation 

The qPCR analysis showed a singular amplicon of 81.5 °C melting temperatures (TM), according to primers RI/f-r, specific for *R. irregularis*, while the primers FM/f-r, amplify for a singular amplicon of 80.5 °C TM, specific for *F. mosseae*. Sequence analysis of the PCR amplicons indicated as isolates S1_V1 (NCBI accession number MK513942) and S5_V3 (MK513943) were homologous to >98% of the reference sequences related to 28S rRNA gene of *R. irregularis*, NCBI accession number HF968988.1, and FJ235574.1. In detail, for *R. irregularis* primers, we identified a 248-bp that included the nucleotide substitutions of A→G; G→A; G→C; A→G; (positions 81; 136; 140; 185; MK513942 and MK513943/ HF968988.1, and FJ235574.1, respectively). Blast analysis of the nucleotide sequences indicated as isolates S4_V1 (MK513940) and S10_V3 (MK513941) revealed a high nucleotide identity with the reference sequences related to the LSU gene of *F. mosseae* (of 98.94% with FN377862.2; 98.42% with FN377865.1) The *F. mosseae* primers identified a 190-bp that included the nucleotide substitutions of A→C (position 68; MK513940 and MK513941/ FN377865.1); CT→GC (positions 116–117; MK513940 and MK513941/FN377865.1 and FN377865.1) (Supplementary Figure S2).

#### 3.3.2. qPCR Assay 

The analysis of the nine replicates of 28SRNA S1_V1 qPCR of target fragments used as template, for the four dilutions used to generate the standard curve on *R. irregularis* ranging from 22.3 + 0.12 quantification Cycle (Cq) for the 3.5 × 10^−8^ ng/reaction, to 31.6 ± 1.1 Cq for the 3.5 ×10^−11^ ng/reaction. Really, the probability of replicates’ detection of the last dilution was lower than 95%, underlining the limit of detection of the gene (Table 1). Similar results were detected for *F. mosseae*. The four tested dilutions related to the LSU gene of the S10_V3 qPCR fragment, ranging from 23.8 ± 0.12 Cq (2.8 × 10^−8^ ng/reaction), to 32.8 ± 0.6 Cq (2.8 × 10^−11^ ng/reaction) (Table 1). The qPCR of both *R. irregularis* and *F. mosseae* serial dilution of target fragments spiked with DNA roots of oak nonhost plants of AMF, at concentrations 50 ng/reaction, was inhibited, while the addition of 5 ng/reaction of DNA from nonhost root showed no inhibition of the PCR amplification (Table 1).

The analyses of four-point serial dilutions related to I10 and A7 grapevine samples root indicate a total inhibition of qPCR amplification testing 100 and 50 ng DNA/reaction for both species, while a positive sample was detected at 5 and 0.5 ng DNA/reaction but not for all replicates (Supplementary Table S1).

#### 3.3.3. ddPCR Assay

**Figure 5:**
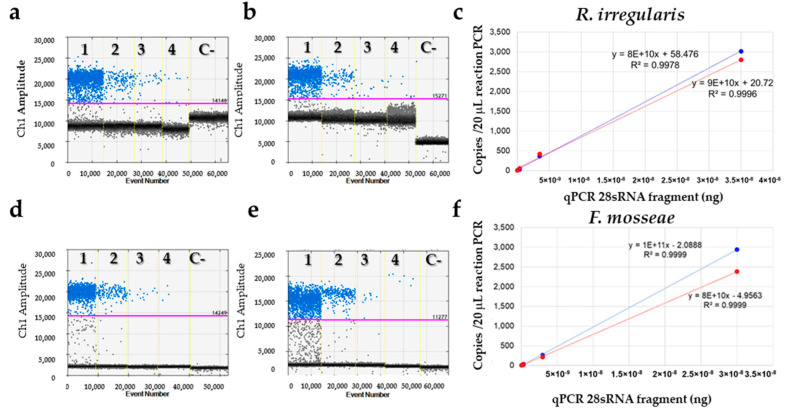
Performance of primer sets with various serially diluted targets in ddPCR. (**a**,**b**,**d**,**e**), The ordinate scales indicate fluorescent amplitude. The pink line is the threshold, above which are positive droplets (blue) containing at least one copy of target DNA and below which are negative droplets (gray) without any target DNA. Samples are divided by the vertical dotted yellow line. (**a**) Ten-fold serial dilutions of *R. irregularis* S1_V1 qPCR fragment (from 3.5×10^−8^ to 3.5×10^−11^ ng/reaction); (**b**) 50 ng/reaction of AMF nonhost DNA from *Quercus ilex*, oak spiked with ten-fold serial dilutions of S1_V1 qPCR fragment; (**c**) linear regression of serial dilutions analyzed in (**a**) (blue line) and (**b**) (red line); (**d**) ten-fold serial dilutions of *F. mosseae* S10_V3 qPCR fragment (from 2.8 × 10^−8^ to 2.8 × 10^−11^ ng/reaction); (**e**) 50 ng/reaction of AMF nonhost DNA from *Quercus ilex* L., oak, spiked with ten-fold serial dilutions of S10_V3 qPCR fragment; (**f**) linear regression of serial dilutions analyzed in (**d**) (blue line) and (**e**) (red line). C- = negative control.

The amount of the droplets was >of 15,000 in 72% of the analyzed samples, and in the range 10,000–15,000 in the 28% for the remainder. Samples relative to the four 10-fold dilutions of 28SRNA and LSU qPCR fragment (analyzed alone or spiked with 50 ng of DNA from nonhost root) showed a good degree of linearity (R2 > 0.996) in the range of about 8 to 2940 copies/20 μL/PCR reaction for *R. irregularis* species and about 5 to 3020 copies/20 μL/PCR reaction for *F. mosseae* (Figure 5d–f; Supplementary Table S2). No ddPCR inhibition was detected related to nonhost DNA added to the reaction. 

**Figure 6:**
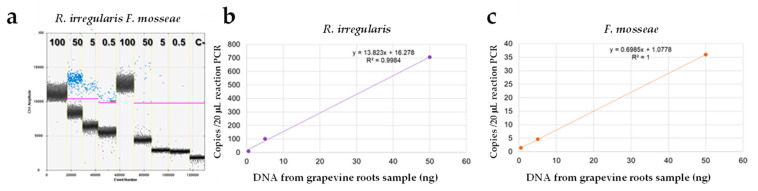
Several dilutions (100, 50, 5, 0.5 ng) of DNA from sample I10 analysed for *R. irregularis* and *F. mosseae* genes (**a**). The leftmost ddPCR reaction (100 ng) was saturated by an excess target concentration and the rightmost reaction contains a minimal copy of the target. The respective regression line of serial dilution was shown (**b**,**c**). C- = negative control.

In the same way, a ten-fold serial dilution performed according to the DNA from I10 and A7 roots grapevine samples showed a good degree of linearity, (R2 > 0.996) for both *R. irregularis* and *F. mosseae*, with detection from 50 to 0.5 ng DNA of roots/reaction, while the amounts of DNA from roots per reaction showed saturation reached at 100 ng of DNA (Figure 6a,b). In the sample I10, from 50 to 0.5 ng DNA of roots/reaction, the quantity of *R. irregularis* target gene ranging from about 706 to 9 copies/20 μL/PCR, while the *F. mosseae* target gene ranged from 36 to 1.4 copies/20 μL/PCR. In sample A7, the quantity of *R. irregularis* target genes ranged from about 105 to 2.2 copies/20 μL/PCR, and the *F. mosseae* target gene from 50 to 1.2 copies/20 μL/PCR (Supplementary Table S3). No amplification was observed according to DNA from grapevine leaf tissue (data not shown).

**Figure 7:**
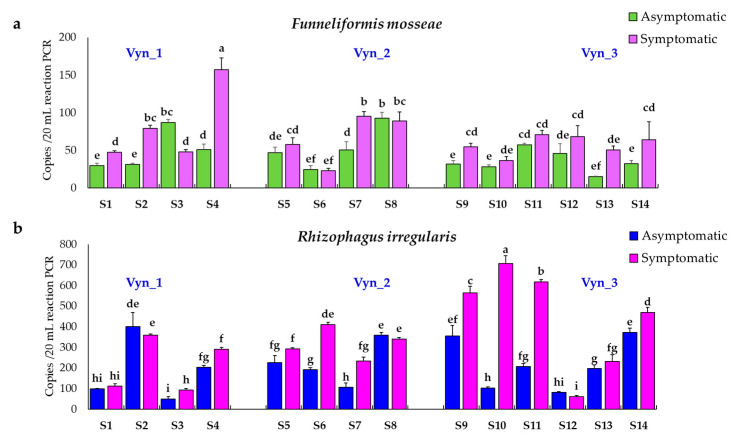
Amount of *F. mosseae* (**a**) and *R. irregularis* (**b**) quantified by ddPCR in each asymptomatic and contiguous esca symptomatic plants from each site (S) individuate in the vineyards (Vyn_1, Vyn_2 and Vyn_3). For each trait, means followed by at least one common letter are not significantly different, according to Duncan’s Multiple Range Test (*p* < 0.05). The test was assessed on two independent experiments for each subsample (n = 4).

Concerning Vyn_1, in esca asymptomatic plants, the abundance of *F. mosseae* ranged from 29.5 ± 3.53 copies/20 μL (20 μL reaction was related to 50 ng DNA of grapevine roots) (plant A1), to 87 ± 4.2 copies/20 μL (plant A3), while in symptomatic plants from 47.5 ± 2.12 copies/20 μL (I1 plant), to 157 ± 15.5 copies/20 μL (I4 plant), were detected (Figure 7a). As for *R. irregularis*, in the roots from esca asymptomatic plants, the quantity ranged from 49 ± 11.3 copies/20 μL (A3 plant), to 400 ± 69.8 copies/20 μL (A2 plant), while in the roots from symptomatic plants the amount ranged from 92.5 ± 7.7 copies/20 μL (I3 plant), to 358 ± 9.9 copies/20 μL (I2 plant) (Figure 7b). Overall, the average copies of *F. mosseae* gene on Vyn_1 were 49.6 ± 4.1 copies/20 μL on asymptomatic plants, and 82.8 ± 5.1 copies/20 μL on symptomatic plants, respectively, while the corresponding numbers for the *R. irregularis* gene were 188.6 ± 22.6 copies/20 μL on asymptomatic plants and 213 ± 9.1 copies/20 μL on symptomatic plants, respectively. 

In Vyn_2, the abundance of *F. mosseae* ranged from 24.5 ± 4.5 copies/20 μL (A6 plant), to 92.5 ± 7.7 copies/20 μL (A8 plant), in esca asymptomatic plants, and from 22.7 ± 3.3 copies/20 μL (I6 plant), to 95.7 ± 6.3 copies/20 μL (I7 plant) in symptomatic plants (Figure 7a). Regarding *R. irregularis*, from 105.5 ± 21.2 copies/20 μL (A7 plant) to 358 ± 14 copies/20 μL (A8 plant), were detected in the esca asymptomatic plants, and from 233.5 ± 19 copies/20 μL (I7 plant), to 410 ± 10.6 copies/20 μL (I6 plant) in asymptomatic plants (Figure 7b). Overall, the average copies of *F. mosseae* gene on Vyn_2 was 53.6 ± 7.6 copies/20 μL on asymptomatic plants, and 66.1 ± 7.5 copies/20 μL on symptomatic plants, while about *R. irregularis* was 200.2 ± 19.7 copies/20 μL on asymptomatic plants and 319.1 ± 10.7 copies/20 μL on symptomatic plants. 

In Vyn_3, the abundance of *F. mosseae* ranged from 15.3 ± 0.3 copies/20 μL (A13 plant), to 57.3 ± 1.8 copies/20 μL (A11 plant) in the esca asymptomatic plants, while it ranged from 36.5 ± 14.8 copies/20 μL (I10 plant), to 71.5 ± 4.9 copies/20 μL (I11 plant) in the symptomatic plants (Figure 7a). Regarding *R. irregularis*, in esca asymptomatic plants the abundance ranged from 81 ± 4.24 copies/20 μL (A12 plant), to 355 ± 50.2 copies/20 μL, (A9 plant), while in symptomatic plants ranged from 61.5 ± 4.9 copies/20 μL (I12 plant), to 706 ± 37.4 copies/20 μL (I10 plant) (Figure 7b). Overall, the average number of copies of the *F. mosseae* gene on Vyn_3 was 37.9 ± 4.9 copies/20 μL on asymptomatic plants and 55.5 ± 12.3 copies/20 μL on symptomatic plants, while the corresponding numbers for the *R. irregularis* gene were 214.3 ± 14.7 copies/20 μL on asymptomatic plants and 345 ± 18 copies/20 μL on symptomatic plants, respectively.

## 4. Discussion

The management of esca must be integrated with an interdisciplinary approach [1]. Since the esca pathogens often exist in soils before planting [10], the study of rhizosphere microorganisms, such as mycorrhizal fungi, could provide important information to better understand the dynamics and the light equilibrium among pathogens and beneficial communities. In this work, we analyzed the interaction among the native mycorrhizal symbiosis in both asymptomatic and esca symptomatic grapevines. Concerning the 14 sites analysed, the comprehensive approach to determine the roots mycorrhizal status by non-vital staining [52], showed the presence of mycorrhizae symbiosis in all roots analysed (F% value = 100%), underlining the ancestral affinity of the grapevine with AMF symbiosis. However, the higher AMF colonization according to both intensity of colonization (M%) and abundance of arbuscules (A%) was detected in esca symptomatic plants more frequently than the neighbouring asymptomatic ones, regardless of cultivars or vineyard. This finding highlighted that esca symptomatology affected the relative amount of total AMF symbiosis in the root system. This was observed despite the high AMF abundance variability detected among the plants inside the vineyards, probably linked to the influence of soil conditions and the rhizosphere affecting AMF symbiosis establishment variability [53,54]. The evidence of the highest overall AMF colonization being present in esca symptomatic plants was supported by the OTUs investigation, which showed higher amounts of ITS sequences belonging to *Glomeromycota* phylum, which includes all AMF species [11], in the roots of symptomatic versus asymptomatic plants.

To analyze if this result was AMF species-specific, we have set up a method to quantify the native species among those commonly associated with the roots of the grapevine as *R. irregularis* and *F. mosseae* species [26,27,55] using ddPCR technology. This technique showed higher sensitivity and specificity, compared to qPCR, for the diagnosis of AMF infection on root DNA, usually contaminated by high content of humic acid and root exudates, which result in PCR inhibition [56]. In our work, both *R. irregularis* and *F. mosseae* were detected in all analyzed plants. Although a great variability was found in the vineyards, the species *R. irregularis* is more often more present than *F. mosseae*. However, our data suggest higher gene copy numbers of both species in symptomatic plants than asymptomatic ones for most of all the analyzed sites or vineyards, but these numbers were not necessarily statistically significant for all cases. AMF in nature may differ in the amount of benefit they provide to hosts, but it is impossible to indicate any single partner as universally ‘beneficial’ or ‘not beneficial’ given the elevated complexity of interactions and conditions implicit in microorganism community. Our work highlights that there was no species-specific relationship among AMF and symptomatic or non-symptomatic esca plants, but that the highest amount of AMF symbiosis observed in symptomatic plants involves the whole mycorrhizal community and their rhizosphere relationship.

New studies are now required to understand what appears to be a paradox. Usually, clear relationships between root-associated fungal communities’ composition and plant health status were demonstrated in several plant-mycorrhizae-pathogen interactions involving both woody perennial crops and annual crops [21,37,57,58,59]. Studies comparing different fungal species highlighted that the degree of protection is highly dependent on the AMF inoculated [60]. In addition, some research pointed out a higher protector effect of *F. mosseae* in comparison to other AMF species [61,62]. However, some studies reported higher severity of symptoms caused by viruses [35,63] or fungi disease [64], on leaves of mycorrhizal plants compared to non-mycorrhizal control plants. 

Concerning grapevine trunk diseases, the inoculation of the roots with the *R. irregularis* has reduced both the number of root lesions and the severity of diseases caused by black foot disease pathogens [65]. AMF have been shown to increase tolerance of grapevine rootstocks to black foot disease caused by *Ilyonectria* spp., and changes in the function of the rhizosphere microbial community [31]. However, interestingly, recent greenhouse studies have shown that in the grapevine rootstocks inoculated with both *I. liriodendra,* a weak pathogen known for causing black foot disease, and *R. irregularis* AMF species, the amount of both AMF and pathogen were highest, unlike the results seen in control plants inoculated with one of the two. Further, the presence of the mycorrhizal fungus did not enhance the physiological parameters of the infected plants [32]. By contrast, recently, in grapevines planted in pots using soil collected from a commercial grapevine nursery, the enrichment of AMF with lowest presence of black-foot pathogens under extreme conditions of water deficit, was detected [38]. These results underline the delicate balance between inhibiting pathogens and allowing entry to beneficial fungi, in particular in esca, whose complexity lies notably in the plurality of the involved fungal pathogens, which are associated with specific symptomatology and epidemiology, from the nursery to the vineyard [66]. Similarly, direct analysis of soil-grown plants can help reveal the how this relationship changes in response to developmental, and/or biotic, and abiotic signals. Some recent work focused on trunk disease-affected vines highlighted the importance of the whole microbial community and fungal endophytes for influencing esca [67,68,69]. On the other hand, there is growing evidence that AMF may not be universally beneficial against pathogens, particularly in natural settings [70]. Then, the increase in mycorrhizal symbiosis on symptomatic plants is therefore not surprising, but our study emphasizes that a different relationship could be established by native AMF species compared to exogenous species. The native AMF composition is a pool of species not foreign to the rhizosphere, compared to that established by applying exogenous mycorrhizal inoculum [71]. On the other hand, it is known that both plant mutualists and pathogens share common molecular and cellular mechanisms for colonizing their hosts [72,73,74]. About native mycorrhizal species, the host plant and other rhizosphere organisms recognize certain organisms as their own and do not activate defense mechanisms. Then, the lack of AMF protection may be due to the relatively stress-free growing conditions among the AMF native microorganisms, which are already essential part of the plants, and the pathogens that occurred in advance in the plants. Therefore, native mycorrhizal symbiosis is useful as a bioindicator of environmental stresses [13,38]. The relationship established between the plant and native mycorrhizal species naturally present in the soil, requires a continuous exchange of signals between the host’s roots and symbiont fungi [13,14]. Among these, an altered pattern of root exudation could affect the plant–mycorrhizae–pathogen relationship [75]. In esca symptomatic plants, wood vascular necrosis or black streaking with black gummy exudates occurs, which also acts at a distance from the place of production [76]. Other works suggest that changes in the root exudates from mycorrhizal plants are partially involved in the susceptibility of these plants to soil-borne microorganisms. Therefore, while the root exudates of mycorrhizal strawberry plants have reduced the sporulation of *Phytophthora fragariae* [77], the root exudates of mycorrhizal potato plants have increased the hatching of the nematodes [78]. In mycorrhizal tomato plants, the above were reported to increase the germination of *Fusarium oxysporum* conidia [79]. Then, this suggests that the pathogen establishment changes the relationship between the plant and all mycorrhizal symbiotes. Esca induces important changes in the root system, including a reduction in root biomass and root hairs. Although it is surprising that a greater presence of beneficial fungi is associated with plants that have reduced their vigor, this condition could further increase the need of the plant to establish mycorrhizal symbiosis useful for its nutritional needs. It is conceivable that pathogens take advantage of this symbiosis program to gain access to the host plant’s resources [80]. A synergistic effect has been described among multiple pathogens when reintroduced into plants [81]. Something similar could also happen with non-pathogenic AMF. They could co-exist or even provide indirect benefits. For instance, AMF could allow to pathogens’ further proliferation as a result of the increased carbon sink typical of AMF [82]. Then, several studies are necessary to understand the reason for this change in AMF community linked to esca symptoms on grapevine.

## 5. Conclusions

This investigation carried out on natural mycorrhizal state of plants affected by esca strengthens the knowledge on how the diseased plant interacts with beneficial microorganisms, modifying their concentration. In synthesis, the analysis of the AMF native symbiosis in grapevine roots showed higher levels of total mycorrhization in the symptomatic plants than in the asymptomatic plants. However, the protocol developed in this work according the ddPCR technology emphasizes that this difference is not associated with individual mycorrhizal species, but instead the entire AMF community contributes to this result. 

In this work we have focused on the esca symptoms–AMF relationship in grapevine roots. The investigation of entire microbial community involved in the grapevine roots, symptomatic and asymptomatic to esca, will be able to clarify many aspects. This work contributes to a better understanding of the interactions between native AMF and the fungi related to esca, and it underlines the role of mycorrhizae as bioindicators of the soil changes taking place in the rhizosphere. These results underline that the interactions between plants and their rhizosphere microbial communities are an important point to consider for the development of strategies for the sustainable management of grapevine esca disease.

## Figures and Tables

**Table 1:** The qPCR inhibitors and limits of quantification estimated by qPCR standard curve. DNA of AMF nonhost roots from oak plants (0, 5 and 50 ng/qPCR reaction) spiked with serial dilutions AMF qPCR fragments (S1_V1 sample, for *R. irregularis,* S10_V3 sample and *F. mosseae*, respectively). The experiments were assessed in triplicate over three independent experiments (n = 9). Cq, quantification cycle; SD, standard deviation; na, not amplified. *, four replicates amplified of nine performed.

		qPCR		
		DNA from Oak Nonhost Plant		
	AMF qPCR Fragments from Grapevine Roots (ng/reaction) +	0 ng	5 ng	50 ng
		Cq mean ± SD		
*R. irregularis*	3.5 × 10^−8^	22.3 ± 0.12	22.9 ± 0.2	na
	3.5 × 10^−9^	25.7 ± 0.16	25.9 ± 0.2	na
	3.5 × 10^−10^	29.4 ± 0.3	30.6 ± 0.3	na
	3.5 × 10^−11^	31.6 ± 1.1 *	32.1 ± 1.7 *	na
*F. mosseae*	2.8 × 10^−8^	23.8 ± 0.12	23.8 ± 0.12	na
	2.8 × 10^−9^	27.4 ± 0.19	27.2 ± 0.19	na
	2.8 × 10^−10^	31.5 ± 0.9	32.8 ± 1.3	na
	2.8 × 10^−11^	32.8 ± 1.7 *	33.6 ± 2.1 *	na
	Statistics of standard curve performance, mean ± SD			
*R. irregularis*	Slope	3.155 ± 0.02	3.162 ± 0.03	na
	Efficiency	107.1 ± 0.74	109.7 ± 0.99	na
	Y-intercept	1.078 ± 0.04	3.97 ± 0.02	na
	Value of fit (R2)	0.99 ± 0.001	0.98 ± 0.002	na
*F. mosseae*	Slope	3.117 ± 0.02	3.092 ± 0.02	na
	Efficiency	109.3 ± 0.87	111.7 ± 1.01	na
	Y-intercept	2.723 ± 0.12	2.495 ± 0.31	na
	Value of fit (R2)	0.98 ± 0.001	0.99 ± 0.002	na

## Data Availability

Sequence data are available in a publicly accessible repository, as reported in the body of the paper, and in Supplementary Figure S1 and Tables S1–S3.

## Supplementary Materials

The following are available online at www.mdpi.com/article/10.3390/jof7100869/s1, Supplementary Figure S1: Symptoms taken into account for the esca disease classification on Verdicchio cultivar. Leaves presented the tiger-striped pattern, with a typical yellow band between the green and necrotic tissues (left) and plant with portion of the canopy and clusters desiccated (central). On the right, canopy of a healthy plant. Supplementary Figure S2: Sequence similarity searches using NCBI Blast web site of AMF *R. irregularis* and *F. mosseae* qPCR fragments. (**a**), Blast alignment according 28SRNA gene of sample S1_V1 (NCBI accession number, MK513942) and S5_V3 (MK513943) showing high nucleotide similarity to *R. irregularis* species (HF968988.1 and FJ235574.1), (**b**) Blast alignment of according to LSU gene of sample S4_V1 (MK513940) and S10_V3 (MK51394) homologous to *F. mosseae* species (FN377862.2 and FN37865.1). Supplementary Table S1: Serial dilutions of DNA from I10 and A7 samples analyzed on qPCR for *R. irregularis* and *F. mosseae* genes. The experiments were assessed in triplicate over three independent experiments (n = 9). Cq, quantification cycle; SD, standard deviation; * less of four replicates amplified of six performed; na, not amplified Supplementary Table S2: The ddPCR inhibitors investigation and LOD for AMF. Non host roots from oak plants (0, and 50 ng/qPCR reaction) spiked with serial dilutions of AMF qPCR fragments (S1_V1 and S10_V3 for *R. irregularis* and *F. mosseae*, respectively) at different concentration. The test was assessed on two independent experiments (n = 2). Supplementary Table S3: Serial dilutions of DNA fromI10 and A7 samples analyzed on ddPCR with the primers for *R. irregularis* gene and LSU *F. mosseae* genes. The test was assessed over two independent experiments (n = 2). SD, standard deviation; na, not amplified.
