# Supplementary material for: The Mycorrizal Status in Vineyards Affected by Esca
Source: J Fungi (Basel). 2021 Oct 16;7(10):869. doi: 10.3390/jof7100869 (PMC8540504; doi:10.3390/jof7100869)
Supplement: Supplementary file 1 [file jof-07-00869-s001.zip › jof-1378546-supplementary.pdf]

Supplementary Figure S1 – Symptoms taken into account for the esca disease classification on Verdicchio cultivar. Leaves presented the tiger-striped pattern, with a typical yellow band between the green and necrotic tissues (left) and plant with portion of the canopy and clusters desiccated (central). On the right, canopy of a healthy plant.

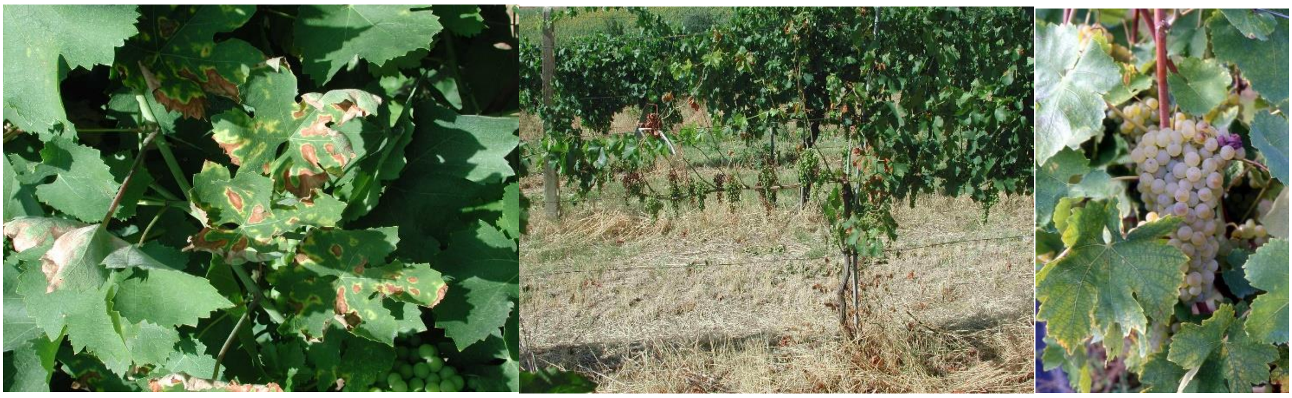

Supplementary Figure S2 – Sequence similarity searches using NCBI Blast web site of AMF *R. irregularis* and *F. mosseae* qPCR fragments. (**a**), Blast alignment according 28SRNA gene of sample S1_V1 (NCBI accession number, MK513942) and S5_V3 (MK513943) showing high nucleotide similarity to *R. irregularis* species (HF968988.1 and FJ235574.1), (**b**) Blast alignment of according to LSU gene of sample S4_V1 (MK513940) and S10_V3 (MK51394) homologous to *F. mosseae* species (FN377862.2 and FN37865.1).

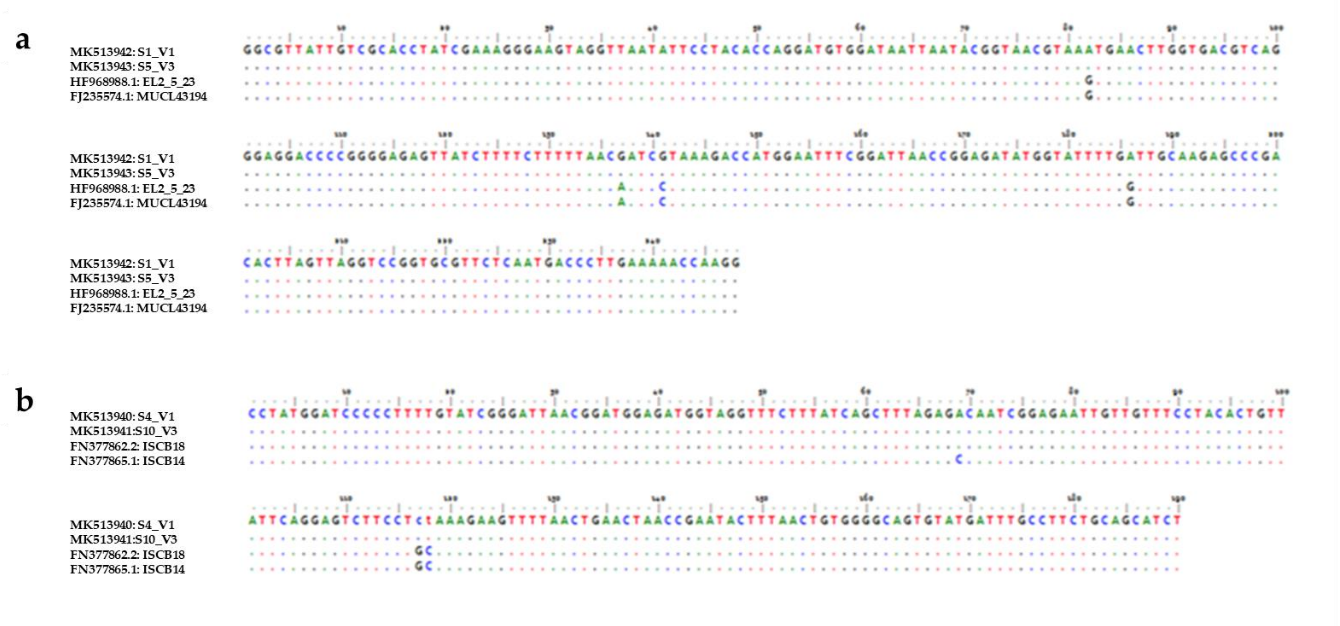

Supplementary Table S1 - Serial dilutions of DNA from I10 and A7 samples analyzed on qPCR for *R. irregularis* and *F. mosseae* genes. The experiments were assessed in triplicate over three independent experiments (n = 9). Cq, quantification cycle; SD, standard deviation; *less of four replicates amplified of six performed; na, not amplified

			qPCR		
			**DNA from grapevine roots (ng)**		
	**sample**	**100**	**50**	**5**	**0.5**
				Cq mean ± SD	
R. irregularis	I10	na	na	29.6 ± 0.3	33.6 ± 1.2*
	A7	na	na	30.2 ± 0.5	35.1 ± *
F. mosseae	I10	na	na	31.2 ± 1.1*	na
	A7	na	na	33.9 ± 1.1*	36.1 ± *

Supplementary Table S2 - The ddPCR inhibitors investigation and LOD for AMF. Non host roots from oak plants (0, and 50 ng/qPCR reaction) spiked with serial dilutions of AMF qPCR fragments (S1_V1 and S10_V3 for *R. irregularis* and *F. mosseae*, respectively) at different concentration. The test was assessed on two independent experiments (n = 2).

		**ddPCR**	
	**AMF qPCR fragments from grapevine roots (ng/reaction) +**	**DNA from oak non-host plant**	
		**0 ng/reaction**	**50 ng/reaction**
		**Concentration (copies/20 L reaction)** Mean ± SD	
R. irregularis	3.5×10^{-8}	2,940 ± 84.1	2,380 ± 90.5
	3.5×10^{-9}	270 ± 32	216 ± 34
	3.5×10^{-10}	38 ± 4.1	24 ± 7.1
	3.5×10^{-11}	10 ± 3.3	8 ± 3.5
F. mosseae	2.8×10^{-8}	3,020 ± 58.3	2,800 ± 68.3
	2.8×10^{-9}	364.2 ± 14	424 ± 19
	2.8×10^{-10}	30 ± 9.0	56 ± 12
	2.8×10^{-11}	5.6 ± 1.1	9.4 ± 21

Supplementary Table S3 - Serial dilutions of DNA fromI10 and A7 samples analyzed on ddPCR with the primers for *R. irregularis* gene and LSU *F. mosseae* genes. The test was assessed over two independent experiments (n = 2). SD, standard deviation; na, not amplified.

		**qqPCR**			
		**DNA from grapevine roots (ng)**			
	**sample**	**100**	**50**	**5**	**0.5**
		**Concentration (copies/20µL)**			
R. irregularis	I10	na	706 ± 80.4	101 ± 21.1	9 ± 2.1
	A7	na	105.4± 21.7	12.4 ± 6.1	2.2 ± 1.8
F. mosseae	I10	na	36 ± 2.82	4.6 ± 2.1	1.4 ± 0.04
	A7	na	50.4 ± 10.7	6.4 ± 3.1	1.2 ± 1.1
